# Experimental, Pathologic and Vivisectional Proof, That “Cancer” Incipiently Is a Localized Chemico-Hyper-Stimulated Toxic Lymph Process

**Published:** 1914-09

**Authors:** Frederick Gaertner

**Affiliations:** Pathologist, Pittsburgh, Pa., U. S. A.; 4129 Penn. Ave.


### BUFFALO MEDICAL JOURNAL

Yearly Volume 70. SEPTEMBER, 1914 No. 2

### ORIGINAL ARTICLES

The right is reserved to decline papers not dealing with practical medical and surgical subjects, and such as might offend or fail to interest readers. Contributors are solely responsible for opinions, methods of expression and revision of proof.

### Experimental, Pathologic and Vivisectional Proof, that "CANCER" Incipiently is a Localized Chemico-hyperstimulated Toxic Lymph Process.

"The greater its toxicity and alkalinity; the greater its colloidal nitrogen stage and the greater its malignancy."

By FREDERICK GAERTNER, A. M., M. D., & LL.D., Etc.

Pathologist, Pittsburgh, Pa., U. S. A.

In 1882, I discovered under Virchow that the "leucocytes" would swallow up foreign elements that occurred in the bloodstream, and in 1885 under Von Recklinghausen, further demonstrated that also the fixed body cells, e.g., the flattened epithelial cells of the lung alveoli; then the parenchyma liver cells and spleenpulp cells would also take up foreign elements; but it was not until 1900 that I conclusively demonstrated vivisectionally and pathologically that the endothelial and especially epithelial cells, i.e., when biochemically stimulated, possess phagocytic propensities; and it is here where we find the pre-etiological factor for arterioscleroses, fatty degenerations even calcifycations; these various pathologic infiltrations, together with experimental inoculative injections of diverse tissue juices, both of acid and alkaline reactions, especially of fermented nitrogenized lymph-juices, even cancer fluid, injected into healthy non-cancerous animals. i.e., the

My first papers, entitled: Experimental Investigations and Pathological Researches On The Cause of Cancer, its intra cellular Pathology, produced by a nitrogenized auto-intoxicated-lymph, were read and fully discussed before the American Association of Clinical Research at Boston, Mass., September 28, 1911, and published by St. Louis Medical Review, Oct., 1911, and Virginia Medical Semi-Monthly, November, 1911. Also by the official organ of A. A. of Clinical Research, The Lancet-Clinic of Cincinnati, January, 1912.

mamma of rats and mice, I could stimulate irregular cell proliferations which were decidedly neoplastic; this eventually led me to make the discovery that cancer can be produced artificially; then I went one step further and demonstrated in the human that slow growing non-malignant tumors, can be made malignant and fast growing by injecting toxinized lymph juices, especially certain chemical stimulants directly into the tumor mass, such as nicotine, cocaine, arsenic and the nitrates; whereas on the other hand, a fast growing malignant cancer could materially be checked up in growth and practically reduced to a dead-cancer or post malignant growth by cutting off its toxic lymph nourishment.

In the above experiments, together with other pathologic observations; no matter how incipient the cancer was, I invariably found outside old scars, lacerations or other irritating traumas; the lymph circulation highly engorged, stagnant and pathologically disorganized with increased interstitial osmotic pressure, by extracting some of this toxinized lymph, which was mucilagenous, highly alkaline with a high percentage of nitrogen; by injecting this same semi-liquid into other non-cancerous animals, I could again produce the characteristic cancer cells, e.g., the greater its toxicity and alkalinity, the greater its colloidal nitrogen stage; and the greater its malignancy.

Since I have produced cancer artificially 1911, and especially since I have transplanted sarcomatous tissue, with its toxic lymph extract into the mammary gland and produced an exquisite cancerous growth, 1913; corroborates conclusively the following six salient points:

First: Cancer is not parasitic; otherwise all would become cancerous that come in contact with it; therefore, non-infections and non-acidulous.

Second: Cancer originates from the interior of the preexisting cells, i.e., from bio-chemically stimulated hyper-alkaline toxic lymph.

Third: Cancer is not hereditary, as it can be produced artifically; some animals are prone to it; it can be stained in situ; "ante-mortem."

Fourth: Cancer could not be a degenerative process but a regenerative retro-progressive one.

Fifth: Cancer is embryonic only in one respect, i.e., that it being a chemic-nitrogenous lymph process.

Sixth: In transplanting cancer masses, its growth can be assured by feeding it with cancer fluid, or artifically prepared nitrogenized toxic lymph, even the sap of vegetation.

**What is cancer?** When are tissues cancerous; can a line of demarcation be drawn between normal and cancer- cells? These questions are fully answered within.

Cancer is purely a lymph process; it is started within the lymph spaces and capillaries; nourished by an intoxicated, alkalinized lymph; carried and metastatized within colloidal nitrogen lymph masses. It can never originate in tissues minus lymphatics, nor develop where the lymphatics have been obliterated, but the more abundantly the tissues are supplied with lymph spaces and capillaries, the more frequently cancer develops.

The original stimulus of embryonal development outside of the union of the two original elements; ovum as cell and spermatazooa as nucleus, is nitrogen lymph; should, however, this process become over stimulated and infiltrated with hypernitrogenously intoxicated lymph; chorio-epithelioma may result, but neither embryonic nor carcinomic tissue can develop in an acid media, inasmuch that the metabolistic activity of normal-cells is acidulous, whereas the neoplastic proclivity of cancer-cells is decidedly alkaline, in which nucleinic acid and phosphorus have been alkalinized into nucleinate of sodium and potassium.

Normal lymph or tissue juices are a poor medium for tissue growth, e.g.,—"the addition of alkaline sodium salts promotes tissue growth, cicatrization and fibrosis (Carrel); whereas the further addition of hypernitrogen stimulation produces regrowth, new-growth and neoplastic proliferations, (Gaertner). This speaks conclusively against the parasitic origin of cancer, however, I have found all kinds of bacteria even maggots in ulcerated cancers, but they have had nothing to do with its original cause, if they did, then all tumors would likewise be of parasitic origin and ushered in by chills and fever, which is absolutely not the case.

I have examined and traced down the histories of thousands of cancer subjects with the view of finding in each its probable cause, in nearly every instance, I found invariably there existed apriori; a pre-cancerous condition followed by a cancerous state, then a pre-malignant one and finally a malignant stage, e.g., aposteriori; locally, there pre-existed, certain chemic-pathological tissue changes, especially the lymph within the obstructed lymph-spaces, and that there occurs sooner or later, a decomposition with disorganization of this stagnated lymph into a hyper-alkaline toxic-lymph. Cancer, like all tumors, originates from the interior of the per-existing cells, muscle-fiber, connective-tissue, spindle-shaped and round cells, even lymphocytes and leucocytes, all are concerned in the formation of tumors, i.e., the result of intrinsic biochemical stimulations and its type of malignancy can be determined by the degree of such pathologic stimulations which produces the greatest amount of retromorphosed endogenous tissue, and to what extent the peri, and subtumoral masses are infiltrated with colloidal nitrogen lymph, toxinized lymph elements, necrosed cancer debris, i.e., chromatin residues, cell remnants, nuclear and neo-proto-plasmic fragments.

Cancer cells are not born, neither are they produced directly from the normal cells, neither have they any function nor do they possess any intrinsic resistance, i.e., total loss of osmotic tension; therefore, prone to absorb more readily than the normal cells. It is admitted that cancer cells originally came from the fixed body cells, but they must pass through various successive stages of intra-cellular morphological, retro-progressive developments; i.e., from a karyakinetic segmentation to a proliferation, then from an abnormal proliferation to a sprouting process.

It is an acknowledged fact that protoplasm (bio-proteine, Muller) physiologically possesses assimilatory and osteogenetic properties of tissue regeneration, its chemical base is nucleinic acid, which has a tendency of excreting large quantities of nitrogen and organic phosphorus, (pathologically, even of earthy phosphates in animals of inanition. If nucleinic acid is capable of inhibiting this elimination, that is to say, of preventing the reabsorption and waste of the most valuable of the intracellular elements, then a total loss of nucleinic acid necessarily changes its functional activity and reproductive regeneration to a pathological proliferation, e.g., diminished osmotic tension. This pathologically elaborated lymph in loco permeates and disorganizes the fixed body cells also penetrates and stimulates the movable cells into hyper-activity. It simmers down to be nothing else, than a pathologically disintegrated but nitrogenously alkalinized tissue-waste into an irritating lymph, acting as an excito-stimulant (intrinsically) to the already weakened, on account of loss of its lipoids and nucleinic acid, into a riotously incited tissue cells.

I have divided cancerous growth into four distinct stages—**Pre-cancerous, Cancerous, Pre-malignant and Malignant.**

First:—The stagnation, fermentation and decomposition of the pent-up tissue-waste into an irritating lymph, with a highly nitrogenous auto-intoxication of the epithelium and other cellular elements (a direct exciting stimulus to the nucleus, nucleolus especially protoplasm) disorganization of its intracellular constituents with loss of its lipoids and nucleinic acid, e.g., increased diffusibility due to increased interstitial alkalinized osmotic pressure.

Second:—An acute, irregular cell proliferation into sprouting cancer cells and rapidly into cancer colonies, thereby tumor formation with a tendency of encapsulation and early lymphatic regional infiltrations.

Third:—A breaking down of the over-stimulated and over-distended toxic nitrogenized cancer-cells, due to toxic nutrition and degenerative changes, further disorganization of the stagnated lymph with toxic disintegration of the same into a colloidal nitrogen-lymph or cancer fluid; extensive stasis, ulceration, necrosis into blood vessels and secondary metastases.

Fourth:—Death. From cancer toxeamia, thrombosis and exhaustion.

The causes leading up to a precancerous state are numerous, manifold and oftentimes complicated. The normal lymph with its circulation is to physiologize the tissue cells, i.e., endothelium and epithelium, etc., in their growth, repair and functional activities; and especially to collect and carry off tissue waste; should, however, the lymph become pathologic, i. e., stagnant and infiltrated with larger quantities of disorganized tissue waste, urea and sugar, the result of fermentation and decomposition, with a Sp. Gr. of 1045 to 1065; within the already engorged and obstructed lymph spaces and capillaries due to environmental pathologic destructive tissue changes, such as mechanical obstructive indurations, fibrous bands, lacerations, old adhesions, also infarctions, organic-metastatic emboli, even inorganic metastases and syphilitic infiltrated penetrating gummata, especially chronic irritations due to congestive inflammatory cicatrizial tissue infiltrations, called by me (semi-inflammatorie sub-cicatracosis.)

In comparing the functional activities of normal cells and the tissue juices, i.e., physiologic-chemical reaction, slightly acidulous; to an abnormal pathologic-biochemical (infiltration) stimulation, decidedly alkaline; I find that the greater the density of its interstitial and intercellular tissue juices, the greater its osmotic pressure; and the greater its concentration, the more frequently its particles remain inactive; but the greater its dilution, the more complete the dissociation of the particles and the greater the degree of ionization; in other words, if we withdraw the water to a minimum, the tissues become drier and the combustion of nitrogen increases, therefore, any alteration in osmotic pressure through increase or decrease in density of the fluids of the tissues affects the volume and shape of the cells and especially its intracellular constituents. However, the elimination of uric acid within the tissues; endogenously, it is derived from the metabolism of organ cells in which nucleinic acid is the active solvent of uric acid, and as long as this is physiologically maintained and carried off, cancer can never develope; therefore, the subject of nuclein and protein metabolism is the one most important for our consideration, and for that reason concentrated proteids and albuminoids should be avoided in cancer subjects; nucleins occur in the nuclei of the cells as nucleo-proteins which consists of nucleinic acid and two albumin components one of which splits up readily, the other with difficulty. Nucleinic acid is the active life cell principal; its formula $C_{40,}$ $H_{54,}$ Az.$_{14,}$ $P_{4,}$ & $O_{27,}$ i.e., the specific ferment of normal cellular growth and especially to physiologizise metabolistic activity, it is stored up within the normal cells as a sort of reserve agent and gives to the cellular elements this intrinsic resistance which cancer cells does not possess; e.g., by injecting nucleinic acid into cancer subjects, its growth is materially checked.

The original normal cell, its reticular nucleus that directs and controls the activity of the cytoplasm contains chromatin, plastin, nucleo-protein, calcium, phosphorus, iron, etc. The cytoplasmic reticulum fibers continuous with those of the nucleus are also composed of plastin and calcium, in the reticulum spaces, we have first ordinary proteins; second, nucleo-proteins with phosphoric acid; third, calcium combinations, and fourth, proteins in combinations with fatty acids, i.e., lipo-proteins, the lipoids forming the cohesive cement of its intracellular constituents. Ont of the most important biochemical and pathological changes brought about by this toxic hyper-nitrogen infiltration, producing a disorganization of its intra-cellular constituents which are thrown into a chaotic upheaval, the nucleus shaken off its very foundation, followed by a marked deficiency, i.e., loss of its intrinsic phosphorus and nucleinic acid, replaced with increased alkalinity (sodium and potassium salts), e.g., increased diffusibility; whereby a gradual obliteration of its reticulum and lipoids, with a destructive nucleo-proteid catobolism, retro-morphosed into a bio-synthetine nitrogen-enzyme, which I named neo-proto-plasm, this is particularly noticeable nearest the cell wall and is the first element that shoots through the already weakened and attenuated cell membrane, by which process the cellular (now semi-neoplastic) elements became much enlarged, pale and finely gradular; in some of the cells, the nucleus was still preserved; in others, the nuclei became very much enlarged, some scattered but mostly subdivided while still others have the appearance of being distorted and situated eccentrically and in many instances, it was flattened out into a concave disc, closely applied to the inner cell wall which conclusively proved to be due to an over-production of intracellular gaseous changes, whilst the cellular elements were in process of crenation, some in mitotic developments, other cellular elements rapidly retroprogressed neoplactically, and many of them overdilated from irregular, gaseous vacuoles with pseudo-anaplastic formations thereby formed into an irregular sprouting neoplastic cell proliferation, even giant cells, into cancer colonies, besides a fibrilla network of coagulated lymph in its meshes were lymph cells, degenerated and disintegrated leucocytes, chromatin residues, and other necrosed cellular debris. In further advanced growths, I found that the semi-neoplastic cells which originally had its functions to secrete, rapidly became cancerous and were so changed and modified by its rapid proliferation, e.g., intrinsic disorganization with increased osmotic tension; the further production of a most virulent-toxic-nitrogen lymph, i.e., distinctly colloidal, which affected other cells in their immediate neighborhood even the leucocytes, lymphoid also the spindle-shaped and connective tissue-cells shows increased neoplastic activity.

This colloidal nitrogen lymph, or cancer fluid, is not absorbable by the blood vessels only where necroses have already occured, and this explains the finding of colloidal nitrogen in the urine of cancerous subjects. Its composition, highly alkaline with sodium and potassium salts, predominating; in a nitrogenous glue-like menstrum, readily gives up nitrogen, whereas in-toto, it is neither absorbable, dissoluble nor diffusible, but readly taken up by the injured lymphatics, especially necrosed blood vessels, and it is my opinion that some of this colloidal nitrogen lymph must be carried along with the metastatic masses in order to form metastases.

Cancer, therefore, is non-acidulous growth, and could not develope in an acid media, e.g., in acidosis and hyperacidosis, and for that reason, primary cancer of the kidneys and stomach are extremely rare, with one exception, of its ''Pylorus;'' this anatomic, ''traumatically'' exposed tract being well supplied with blood and decidedly so with lymph and lymphatics; before primary cancer can develop itself in the above organs, certain gross pathological tissue changes, must long have pre-existed, even prior to the precancerous state, such as chronic inflammatory indurations, with necrosis and ulceration, i.e., diminished acidity; however, the finding of certain fatty-acids in its outlying districts of cancer of the intestinal tract, also of bone, which causes a decalcyfication of the latter, prior to cancer infiltrations, are due to intracellular destructive changes in which its lipoids and nucleinic acid were displaced by increased alkalinity; which either neutralysis, the fatty acids, or increases them interstitially, through intercellular and interstitial nitrogenized alkaline accumulations, pushes the fatty acids to one side and further concentrates this toxic lymph into a colloidal nitrogen lymph which, on account of its intense cohesiveness intrinsically, heavier in specific gravity: and less volatile, are retained within the immediate proximity of the growth and thereby supply an increased amount of pathologic lymph.

Pathologists, asked themselves: Why should an atrophied organ, as the mamma and uterus after menopause, abruptly change into a proliferating mass? Accurate microscopic researches at autopsies show remnants of old scars, lacerations, adhesions and cystic formations, then again where the tissues have been traumatized and weakened by continuous mild injuries, i. e., the unnecessary and repeated unskilled operations, whereas in the skin, the result of long continuous chronic irritations in warty growth; chronic eczematous patches; sebaceous tumors; old ulcers; irritating cracked lips and abrasions alae-nasi, however, the precancerous contributive pathological changes in the glandular organs, i. e., the mammary, testes, ovaries, liver, spleen, kidneys, prostate and lymph glands are due to chronic inflammatory enlargements, cysts, semi-indurations from traumatism, old adhesions, and fibrous bands, especially old gonorrheal and syphilitic gumma infiltrations; these may lead to intrinsic proximal pressure, its lymph return becomes pathologically stagnated; fermentative chemical changes are inevitable with the production of a nitrogenized auto-intoxicated lymph.

4129 Penn. Ave.

---

**Resolution of Appendicitis and Salpingitis.** Maurice Peraire and Jean Boyet, Bull. et Mem. de la Soc. de Med. de Paris, May 23, 1914. (The term **refroidissement** is not exactly translated by resolution but refers to a state in which surgical intervention is not urgent.) The authors consider the presence of diaceturia an indication for surgical intervention and its absence quite a reliable justification for palliative methods. The test of Ondrejovich is considered the most convenient for the clinician. Urotropin does not interfere with the test but salicylic derivatives, phenols, phenyl-hydrazin, pyridine, mercurial salts and possibly other substances may. General conditions of metabolism and diet may also interfere but with the liquid diet usually employed, absence of diabetes, excessive intestinal putrefaction indicated by indican and with due caution to avoid conflicting medication which is seldom indicated, these theoretic contraindications to the test are not of much importance. The reagents employed are glacial acetic acid, 2% aqueous solution of methylene blue and tincture of iodine. To 10 c. c. of urine in a test tube are added 3 drops of glacial acetic acid and 2 drops of methylene blue solution. The urine assumes a blue or green tint. 4 drops of tincture of iodine changes the color to red. If the urine contains diacetic acid, the original color returns in a few seconds, one minute being the limit of time adopted in pronouncing whether the test is positive or negative.

